# Bis(oxonium) tetra­kis(*o*-toluidinium) cyclo­hexa­phosphate

**DOI:** 10.1107/S1600536810006537

**Published:** 2010-02-27

**Authors:** Houda Marouani, Mohamed Rzaigui, Salem S. Al-Deyab

**Affiliations:** a Laboratoire de Chimie des Matériaux, Faculté des Sciences de Bizerte, 7021 Zarzouna Bizerte, Tunisia; b Petrochemical Research Chair, College of Science, King Saud University, Riyadh, Saudi Arabia

## Abstract

In the title compound, 4C_7_H_10_N^+^·2H_3_O^+^·P_6_O_18_^6−^, the complete cyclo­hexa­phosphate anion is generated by crystallographic inversion symmetry. In the crystal, the H_3_O^+^ ions and the [P_6_O_18_]^6−^ anions are linked by O—H⋯O hydrogen bonds, generating infinite layers lying parallel to the *ab* plane at *z* = 1/2. These layers are inter­connected by the organic cations, which establish N—H⋯O hydrogen bonds with the [P_6_O_18_]^6−^ anions.

## Related literature

For further synthetic details, see: Schülke & Kayser (1985 ▶). For related structures, see: Amri *et al.* (2008 ▶); Larafa *et al.* (1997 ▶); Akriche & Rzaigui (2000 ▶); Selmi *et al.* (2009 ▶); Khemiri *et al.* (2009 ▶). For a discussion on hydrogen bonding, see: Brown (1976 ▶); Blessing (1986 ▶). For tetra­hedral distortions, see: Baur (1974 ▶).
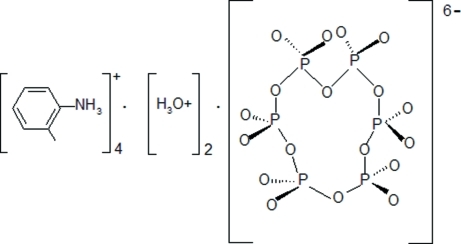


## Experimental

### 

#### Crystal data

4C_7_H_10_N^+^·2H_3_O^+^·P_6_O_18_^6−^
*M* *_r_* = 944.51
Triclinic, 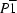

*a* = 9.344 (3) Å
*b* = 10.360 (2) Å
*c* = 11.537 (2) Å
α = 95.35 (4)°
β = 92.23 (3)°
γ = 116.00 (5)°
*V* = 995.4 (4) Å^3^
*Z* = 1
Mo *K*α radiation
μ = 0.36 mm^−1^
*T* = 293 K
0.25 × 0.20 × 0.15 mm

#### Data collection

Enraf–Nonius CAD-4 diffractometer
6038 measured reflections
5773 independent reflections
3061 reflections with *I* > 2σ(*I*)
*R*_int_ = 0.025
2 standard reflections every 120 min  intensity decay: 10%

#### Refinement

*R*[*F* ^2^ > 2σ(*F* ^2^)] = 0.052
*wR*(*F* ^2^) = 0.114
*S* = 1.01
5773 reflections
278 parameters
6 restraints
H atoms treated by a mixture of independent and constrained refinement
Δρ_max_ = 0.33 e Å^−3^
Δρ_min_ = −0.38 e Å^−3^

### 

Data collection: *CAD-4 EXPRESS* (Enraf–Nonius, 1994 ▶); cell refinement: *CAD-4 EXPRESS*; data reduction: *XCAD4* (Harms & Wocadlo, 1996 ▶); program(s) used to solve structure: *SHELXS86* (Sheldrick, 2008 ▶); program(s) used to refine structure: *SHELXL97* (Sheldrick, 2008 ▶); molecular graphics: *ORTEP-3* (Farrugia, 1997 ▶); software used to prepare material for publication: *WinGX* (Farrugia, 1999 ▶).

## Supplementary Material

Crystal structure: contains datablocks I, global. DOI: 10.1107/S1600536810006537/hb5337sup1.cif

Structure factors: contains datablocks I. DOI: 10.1107/S1600536810006537/hb5337Isup2.hkl

Additional supplementary materials:  crystallographic information; 3D view; checkCIF report

## Figures and Tables

**Table 1:** Hydrogen-bond geometry (Å, °)

*D*—H⋯*A*	*D*—H	H⋯*A*	*D*⋯*A*	*D*—H⋯*A*
O10—H110⋯O9	0.86 (1)	1.62 (1)	2.469 (3)	170 (3)
O10—H210⋯O2^i^	0.86 (1)	1.69 (1)	2.550 (3)	177 (3)
O10—H310⋯O6^ii^	0.86 (1)	1.67 (1)	2.524 (3)	171 (3)
N1—H1*A*⋯O8^iii^	0.89	1.86	2.753 (3)	177
N1—H1*B*⋯O2^i^	0.89	1.98	2.853 (3)	168
N1—H1*C*⋯O5^iii^	0.89	1.92	2.800 (3)	169
N2—H2*A*⋯O6^iv^	0.89	2.35	3.085 (4)	140
N2—H2*B*⋯O5^ii^	0.89	2.02	2.904 (3)	176
N2—H2*C*⋯O1^v^	0.89	1.83	2.710 (3)	170

Symmetry codes: (i) 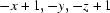; (ii) 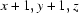; (iii) 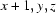; (iv) 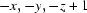; (v) 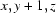.

**Fig. 1.**
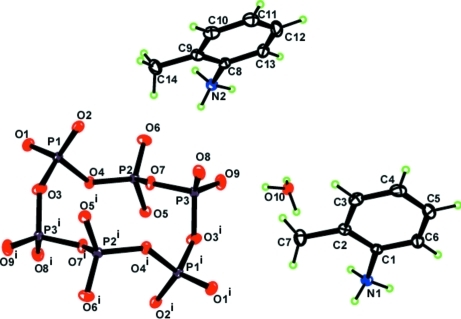
The molecular structure of (I) with displacement ellipsoids drawn at the 30% probability level. H atoms are represented as small spheres of arbitrary radii. Symmetry code: i: -x, -y, -z.

**Fig. 2.**
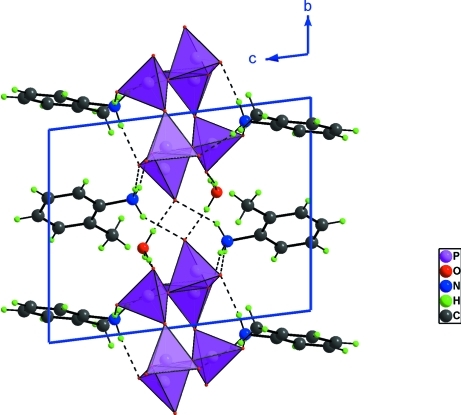
Projection of the structure of (I) along the a axis.

## supplementary crystallographic information

### Comment

Many cyclohexaphosphates of organic cations and inorganic cations (mono, bi and trivalent) have been synthesized and structurally characterized. But the association of the oxonium cation to this kind of material is very rare. On the other hand, there is only one cyclohexaphosphate of mixed cation (organic-oxonium) (Amri, *et al.*, 2008). In this work, we report the preparation and the structural investigation of a new organic oxonium cyclohexaphospohate, (*o*-CH_3_C_6_H_4_NH_3_)_4_(H_3_O)_2_P_6_O_18_, (I).

The title compound is built up from P_6_O_18_^6-^ anion, four organic *o*-toluidinium and two oxonium cations (Fig. 1). The half of the anion, two organic and one oxonium cations constitute the asymmetric unit of (I). The atomic arrangement of the title compound is characterized by the existence of inorganic layers, built by P_6_O_18_^6-^ ring anions and oxonium cations. Each cyclohexaphosphate group is connected to its adjacent neighbours by six oxonium ions through strong O—H···O hydrogen bonds (Table 1) (H···O = 1.66 Å) (Blessing, 1986); (Brown, 1976). The same phenomenon has been observed for (C_10_H_13_NH_3_)_4_(H_3_O)_2_P_6_O_18_.3H_2_O (Amri, *et al.*, 2008).

It is worth noting that the H_3_O^+^ ions exhibit a pyramidal geometry. These layers formed by P_6_O_18_ groups and oxonium ions cross the unit cell parallel to the (a, b) plane at z = 1/2 (Fig. 2). Between these layers, separated by a distance of 11.537 (2) Å, organic cations establish hydrogen bonds to interconnect the different anions. The N(1)H_3_ groups produce the internal P_6_O_18_ ring cohesion through hydrogen bonds involving external oxygen atoms of each PO_4_ tetrahedron. The other N(2)H_3_ groups, link three different rings and so contribute to the interlayer cohesion of this compound. Inside such a structure, the phosphoric ring has an -1 internal symmetry. It develops around the inversion centre located at (0, 0, 1/2), so it is built up by only three independent tetrahedra. The calculated average values of the distortion indices (Baur, 1974) corresponding to the different angles and distances in the PO_4_ tetrahedra [DI (OPO) = 0.038; DI (PO) = 0.039; and DI (OO) = 0.012], show a pronounced distortion of the PO distances and OPO angles if compared to OO distances. So, the PO_4_ group can be considered as a rigid regular arrangement of oxygen atoms, with the phosphorus atom slightly displaced from the gravity centre.

In this atomic arrangement exist two independent *o*-toluidinium cations. Interatomic bond lengths and angles of these groups spread respectively within the ranges [1.367 (5)-1.504 (4) Å] and [115.7 (3)-122.8 (3)°]. The aromatic rings are planar with an average deviation of 0.000189 Å and form a dihedral angle of 28.53°. These values are similar to those obtained for the same organic group in other compounds (Larafa, *et al.* 1997); (Akriche & Rzaigui, 2000); (Selmi, *et al.*, 2009); (Khemiri *et al.*, 2009).

### Experimental

The title compound has been prepared in two steps. In the first one, we prepare Li_6_P_6_O_18_.6H_2_O according to the process described by Schülke and Kayser (Schülke & Kayser, 1985). From this lithium salt, we prepare an aqueous solution of cyclohexaphosphate acid H_6_P_6_O_18_ by passing a solution of Li_6_P_6_O_18_.6H_2_O (5 g in 100 ml) through an ion- exchange resin in its H-state (Amberlite IR 120). In the second step, at 20 ml of the aqueous solution of H_6_P_6_O_18_ freshly prepared, we add drop by drop a solution of *o*-toluidine (30 mmol in 20 ml of ethanol) under continuous stirring.

In order to avoid the hydrolysis of the ring anion the above reaction is performed at room temperature. The so-obtained solution is then slowly evaporated until1 the formation of pink prisms of (I). The title compound is stable for months under normal conditions of temperature and relative humidity.

### Crystal data

**Table:**

4C_7_H_10_N^+^·2H_3_O^+^·P_6_O_18_^6^^−^	*Z* = 1
*M**_r_* = 944.51	*F*(000) = 492
Triclinic, *P*1	*D*_x_ = 1.576 Mg m^−^^3^
Hall symbol: -P 1	Mo *K*α radiation, λ = 0.71073 Å
*a* = 9.344 (3) Å	Cell parameters from 25 reflections
*b* = 10.360 (2) Å	θ = 10–12°
*c* = 11.537 (2) Å	µ = 0.36 mm^−^^1^
α = 95.35 (4)°	*T* = 293 K
β = 92.23 (3)°	Prism, pink
γ = 116.00 (5)°	0.25 × 0.20 × 0.15 mm
*V* = 995.4 (4) Å^3^	

### Data collection

**Table:**

Enraf–Nonius CAD-4 diffractometer	*R*_int_ = 0.025
Radiation source: fine-focus sealed tube	θ_max_ = 30.0°, θ_min_ = 3.0°
graphite	*h* = −13→13
non–profiled ω scans	*k* = −14→14
6038 measured reflections	*l* = 0→16
5773 independent reflections	2 standard reflections every 120 min
3061 reflections with *I* > 2σ(*I*)	intensity decay: 10%

### Refinement

**Table:**

Refinement on *F*^2^	Primary atom site location: structure-invariant direct methods
Least-squares matrix: full	Secondary atom site location: difference Fourier map
*R*[*F*^2^ > 2σ(*F*^2^)] = 0.052	Hydrogen site location: inferred from neighbouring sites
*wR*(*F*^2^) = 0.114	H atoms treated by a mixture of independent and constrained refinement
*S* = 1.01	*w* = 1/[σ^2^(*F*_o_^2^) + (0.0495*P*)^2^ + 0.1583*P*] where *P* = (*F*_o_^2^ + 2*F*_c_^2^)/3
5773 reflections	(Δ/σ)_max_ < 0.001
278 parameters	Δρ_max_ = 0.33 e Å^−^^3^
6 restraints	Δρ_min_ = −0.38 e Å^−^^3^

### Special details

**Table:**

Geometry. All esds (except the esd in the dihedral angle between two l.s. planes) are estimated using the full covariance matrix. The cell esds are taken into account individually in the estimation of esds in distances, angles and torsion angles; correlations between esds in cell parameters are only used when they are defined by crystal symmetry. An approximate (isotropic) treatment of cell esds is used for estimating esds involving l.s. planes.
Refinement. Refinement of *F*^2^ against ALL reflections. The weighted *R*-factor wR and goodness of fit *S* are based on *F*^2^, conventional *R*-factors *R* are based on *F*, with *F* set to zero for negative *F*^2^. The threshold expression of *F*^2^ > σ(*F*^2^) is used only for calculating *R*-factors(gt) etc. and is not relevant to the choice of reflections for refinement. *R*-factors based on *F*^2^ are statistically about twice as large as those based on *F*, and *R*- factors based on ALL data will be even larger.

### Fractional atomic coordinates and isotropic or equivalent isotropic displacement parameters (Å^2^)

**Table:**

	*x*	*y*	*z*	*U*_iso_*/*U*_eq_	
P1	0.19977 (8)	−0.14917 (7)	0.55334 (6)	0.02334 (15)	
P2	−0.14713 (8)	−0.25078 (7)	0.56200 (6)	0.02236 (15)	
P3	0.28988 (8)	0.16136 (7)	0.62215 (6)	0.02600 (16)	
O1	0.2843 (2)	−0.2032 (2)	0.62940 (17)	0.0356 (5)	
O2	0.1635 (2)	−0.2086 (2)	0.42722 (15)	0.0313 (4)	
O3	0.2961 (2)	0.0217 (2)	0.55757 (19)	0.0398 (5)	
O4	0.0375 (2)	−0.1674 (2)	0.60834 (15)	0.0285 (4)	
O5	−0.2360 (2)	−0.2181 (2)	0.65479 (16)	0.0306 (4)	
O6	−0.1935 (2)	−0.40394 (19)	0.51838 (17)	0.0372 (5)	
O7	−0.1447 (2)	−0.1662 (2)	0.45304 (16)	0.0293 (4)	
O8	0.2395 (2)	0.1384 (2)	0.74021 (16)	0.0401 (5)	
O9	0.4383 (2)	0.2884 (2)	0.6012 (2)	0.0453 (6)	
O10	0.7313 (2)	0.3898 (2)	0.64060 (18)	0.0321 (4)	
N1	0.9215 (3)	0.0733 (2)	0.74960 (18)	0.0291 (5)	
H1A	1.0242	0.0929	0.7486	0.044*	
H1B	0.8992	0.1265	0.7023	0.044*	
H1C	0.8609	−0.0201	0.7255	0.044*	
N2	0.4244 (3)	0.6264 (2)	0.6809 (2)	0.0320 (5)	
H2A	0.3811	0.5381	0.6413	0.048*	
H2B	0.5279	0.6711	0.6701	0.048*	
H2C	0.3755	0.6766	0.6555	0.048*	
C1	0.8890 (3)	0.1076 (3)	0.8692 (2)	0.0269 (5)	
C2	0.7377 (3)	0.0942 (3)	0.8895 (2)	0.0303 (6)	
C3	0.7148 (4)	0.1246 (3)	1.0057 (3)	0.0382 (7)	
H3	0.6155	0.1174	1.0236	0.046*	
C4	0.8337 (4)	0.1648 (3)	1.0948 (3)	0.0428 (8)	
H4	0.8136	0.1824	1.1715	0.051*	
C5	0.9816 (4)	0.1791 (4)	1.0705 (3)	0.0453 (8)	
H5	1.0629	0.2084	1.1304	0.054*	
C6	1.0098 (4)	0.1498 (3)	0.9566 (3)	0.0392 (7)	
H6	1.1098	0.1585	0.9394	0.047*	
C7	0.6060 (4)	0.0507 (4)	0.7933 (3)	0.0455 (8)	
H7A	0.6445	0.1115	0.7325	0.068*	
H7B	0.5172	0.0613	0.8240	0.068*	
H7C	0.5724	−0.0484	0.7620	0.068*	
C8	0.4056 (3)	0.6151 (3)	0.8077 (2)	0.0311 (6)	
C9	0.2550 (4)	0.5396 (3)	0.8433 (3)	0.0357 (7)	
C10	0.2453 (4)	0.5348 (3)	0.9631 (3)	0.0445 (8)	
H10	0.1461	0.4832	0.9906	0.053*	
C11	0.3771 (5)	0.6038 (4)	1.0416 (3)	0.0489 (8)	
H11	0.3667	0.5987	1.1211	0.059*	
C12	0.5246 (4)	0.6802 (4)	1.0034 (3)	0.0536 (9)	
H12	0.6139	0.7282	1.0571	0.064*	
C13	0.5409 (4)	0.6862 (3)	0.8859 (3)	0.0414 (7)	
H13	0.6408	0.7370	0.8592	0.050*	
C14	0.1088 (4)	0.4694 (4)	0.7588 (3)	0.0517 (9)	
H14A	0.0923	0.5425	0.7232	0.078*	
H14B	0.0177	0.4154	0.7996	0.078*	
H14C	0.1226	0.4054	0.6995	0.078*	
H110	0.6282 (11)	0.348 (3)	0.633 (3)	0.057 (11)*	
H210	0.770 (4)	0.331 (3)	0.617 (3)	0.088 (15)*	
H310	0.767 (4)	0.464 (2)	0.603 (3)	0.079 (14)*	

### Atomic displacement parameters (Å^2^)

**Table:**

	*U*^11^	*U*^22^	*U*^33^	*U*^12^	*U*^13^	*U*^23^
P1	0.0218 (3)	0.0296 (4)	0.0229 (3)	0.0147 (3)	0.0036 (3)	0.0058 (3)
P2	0.0215 (3)	0.0218 (3)	0.0225 (3)	0.0082 (3)	0.0057 (3)	0.0030 (3)
P3	0.0185 (3)	0.0275 (4)	0.0299 (4)	0.0086 (3)	0.0005 (3)	0.0025 (3)
O1	0.0377 (11)	0.0476 (12)	0.0310 (11)	0.0275 (10)	−0.0009 (9)	0.0066 (9)
O2	0.0350 (10)	0.0473 (12)	0.0210 (9)	0.0265 (9)	0.0047 (8)	0.0049 (8)
O3	0.0369 (11)	0.0293 (10)	0.0565 (14)	0.0154 (9)	0.0238 (10)	0.0090 (9)
O4	0.0213 (9)	0.0425 (11)	0.0216 (9)	0.0142 (8)	0.0045 (7)	0.0026 (8)
O5	0.0236 (9)	0.0344 (10)	0.0308 (10)	0.0099 (8)	0.0107 (8)	0.0020 (8)
O6	0.0477 (13)	0.0247 (10)	0.0369 (12)	0.0136 (9)	0.0109 (10)	0.0026 (9)
O7	0.0198 (9)	0.0364 (10)	0.0312 (10)	0.0102 (8)	0.0027 (7)	0.0139 (8)
O8	0.0379 (11)	0.0550 (14)	0.0246 (10)	0.0191 (10)	−0.0039 (9)	0.0024 (9)
O9	0.0206 (10)	0.0385 (12)	0.0654 (16)	0.0028 (9)	−0.0009 (10)	0.0085 (11)
O10	0.0235 (10)	0.0306 (11)	0.0408 (12)	0.0106 (9)	0.0030 (9)	0.0052 (9)
N1	0.0302 (12)	0.0319 (12)	0.0253 (12)	0.0140 (10)	0.0049 (9)	0.0024 (9)
N2	0.0292 (12)	0.0334 (13)	0.0324 (13)	0.0128 (10)	0.0054 (10)	0.0041 (10)
C1	0.0327 (14)	0.0254 (13)	0.0225 (13)	0.0129 (11)	0.0047 (11)	0.0026 (10)
C2	0.0348 (15)	0.0285 (14)	0.0277 (14)	0.0133 (12)	0.0053 (12)	0.0063 (11)
C3	0.0427 (17)	0.0471 (18)	0.0327 (16)	0.0249 (15)	0.0159 (13)	0.0113 (13)
C4	0.066 (2)	0.0483 (19)	0.0228 (15)	0.0332 (17)	0.0109 (14)	0.0035 (13)
C5	0.052 (2)	0.057 (2)	0.0261 (16)	0.0254 (17)	−0.0059 (14)	0.0004 (14)
C6	0.0339 (15)	0.0509 (19)	0.0329 (16)	0.0194 (14)	0.0021 (13)	0.0026 (14)
C7	0.0324 (16)	0.063 (2)	0.0357 (17)	0.0160 (16)	0.0044 (13)	0.0086 (15)
C8	0.0341 (15)	0.0313 (14)	0.0317 (15)	0.0176 (12)	0.0047 (12)	0.0057 (12)
C9	0.0398 (16)	0.0298 (15)	0.0350 (16)	0.0135 (13)	0.0053 (13)	0.0025 (12)
C10	0.057 (2)	0.0418 (18)	0.0396 (18)	0.0242 (16)	0.0180 (16)	0.0109 (14)
C11	0.069 (2)	0.052 (2)	0.0338 (18)	0.0326 (19)	0.0072 (17)	0.0085 (15)
C12	0.048 (2)	0.071 (2)	0.0391 (19)	0.0263 (19)	−0.0116 (16)	0.0009 (17)
C13	0.0350 (16)	0.0500 (19)	0.0409 (18)	0.0202 (15)	0.0024 (13)	0.0073 (15)
C14	0.0348 (17)	0.049 (2)	0.052 (2)	0.0033 (15)	0.0034 (15)	−0.0024 (16)

### Geometric parameters (Å, °)

**Table:**

P1—O1	1.461 (2)	C2—C3	1.395 (4)
P1—O2	1.491 (2)	C2—C7	1.504 (4)
P1—O3	1.591 (2)	C3—C4	1.374 (4)
P1—O4	1.6075 (19)	C3—H3	0.9300
P2—O6	1.480 (2)	C4—C5	1.367 (5)
P2—O5	1.4830 (19)	C4—H4	0.9300
P2—O7	1.5931 (19)	C5—C6	1.383 (4)
P2—O4	1.594 (2)	C5—H5	0.9300
P3—O8	1.465 (2)	C6—H6	0.9300
P3—O9	1.483 (2)	C7—H7A	0.9600
P3—O3	1.590 (2)	C7—H7B	0.9600
P3—O7^i^	1.6035 (19)	C7—H7C	0.9600
O7—P3^i^	1.6035 (19)	C8—C9	1.378 (4)
O10—H110	0.862 (10)	C8—C13	1.388 (4)
O10—H210	0.863 (10)	C9—C10	1.393 (4)
O10—H310	0.864 (10)	C9—C14	1.496 (4)
N1—C1	1.470 (3)	C10—C11	1.367 (5)
N1—H1A	0.8900	C10—H10	0.9300
N1—H1B	0.8900	C11—C12	1.369 (5)
N1—H1C	0.8900	C11—H11	0.9300
N2—C8	1.489 (3)	C12—C13	1.375 (5)
N2—H2A	0.8900	C12—H12	0.9300
N2—H2B	0.8900	C13—H13	0.9300
N2—H2C	0.8900	C14—H14A	0.9600
C1—C6	1.370 (4)	C14—H14B	0.9600
C1—C2	1.390 (4)	C14—H14C	0.9600
			
O1—P1—O2	118.49 (11)	C4—C3—C2	122.3 (3)
O1—P1—O3	110.27 (13)	C4—C3—H3	118.8
O2—P1—O3	106.30 (12)	C2—C3—H3	118.8
O1—P1—O4	108.90 (11)	C5—C4—C3	120.0 (3)
O2—P1—O4	109.44 (11)	C5—C4—H4	120.0
O3—P1—O4	102.21 (11)	C3—C4—H4	120.0
O6—P2—O5	118.69 (12)	C4—C5—C6	119.7 (3)
O6—P2—O7	108.57 (12)	C4—C5—H5	120.1
O5—P2—O7	110.85 (11)	C6—C5—H5	120.1
O6—P2—O4	111.40 (12)	C1—C6—C5	119.4 (3)
O5—P2—O4	106.38 (11)	C1—C6—H6	120.3
O7—P2—O4	99.21 (11)	C5—C6—H6	120.3
O8—P3—O9	121.31 (13)	C2—C7—H7A	109.5
O8—P3—O3	111.13 (13)	C2—C7—H7B	109.5
O9—P3—O3	107.21 (12)	H7A—C7—H7B	109.5
O8—P3—O7^i^	106.23 (11)	C2—C7—H7C	109.5
O9—P3—O7^i^	107.42 (12)	H7A—C7—H7C	109.5
O3—P3—O7^i^	101.72 (12)	H7B—C7—H7C	109.5
P3—O3—P1	137.49 (13)	C9—C8—C13	122.6 (3)
P2—O4—P1	133.83 (12)	C9—C8—N2	119.2 (2)
P2—O7—P3^i^	129.82 (12)	C13—C8—N2	118.2 (3)
H110—O10—H210	112 (2)	C8—C9—C10	116.4 (3)
H110—O10—H310	110 (2)	C8—C9—C14	122.2 (3)
H210—O10—H310	110 (2)	C10—C9—C14	121.4 (3)
C1—N1—H1A	109.5	C11—C10—C9	122.0 (3)
C1—N1—H1B	109.5	C11—C10—H10	119.0
H1A—N1—H1B	109.5	C9—C10—H10	119.0
C1—N1—H1C	109.5	C10—C11—C12	120.2 (3)
H1A—N1—H1C	109.5	C10—C11—H11	119.9
H1B—N1—H1C	109.5	C12—C11—H11	119.9
C8—N2—H2A	109.5	C11—C12—C13	120.1 (3)
C8—N2—H2B	109.5	C11—C12—H12	120.0
H2A—N2—H2B	109.5	C13—C12—H12	120.0
C8—N2—H2C	109.5	C12—C13—C8	118.8 (3)
H2A—N2—H2C	109.5	C12—C13—H13	120.6
H2B—N2—H2C	109.5	C8—C13—H13	120.6
C6—C1—C2	122.8 (3)	C9—C14—H14A	109.5
C6—C1—N1	118.0 (2)	C9—C14—H14B	109.5
C2—C1—N1	119.1 (2)	H14A—C14—H14B	109.5
C1—C2—C3	115.7 (3)	C9—C14—H14C	109.5
C1—C2—C7	122.8 (3)	H14A—C14—H14C	109.5
C3—C2—C7	121.5 (3)	H14B—C14—H14C	109.5

Symmetry codes: (i) −*x*, −*y*, −*z*+1.

### Hydrogen-bond geometry (Å, °)

**Table:**

*D*—H···*A*	*D*—H	H···*A*	*D*···*A*	*D*—H···*A*
O10—H110···O9	0.86 (1)	1.62 (1)	2.469 (3)	170 (3)
O10—H210···O2^ii^	0.86 (1)	1.69 (1)	2.550 (3)	177 (3)
O10—H310···O6^iii^	0.86 (1)	1.67 (1)	2.524 (3)	171 (3)
N1—H1A···O8^iv^	0.89	1.86	2.753 (3)	177
N1—H1B···O2^ii^	0.89	1.98	2.853 (3)	168
N1—H1C···O5^iv^	0.89	1.92	2.800 (3)	169
N2—H2A···O6^i^	0.89	2.35	3.085 (4)	140
N2—H2B···O5^iii^	0.89	2.02	2.904 (3)	176
N2—H2C···O1^v^	0.89	1.83	2.710 (3)	170

Symmetry codes: (ii) −*x*+1, −*y*, −*z*+1; (iii) *x*+1, *y*+1, *z*; (iv) *x*+1, *y*, *z*; (i) −*x*, −*y*, −*z*+1; (v) *x*, *y*+1, *z*.

## References

[bb1] Akriche, S. & Rzaigui, M. (2000). *Solid State Sci.* **2**, 397–403.

[bb2] Amri, O., Abid, S. & Rzaigui, M. (2008). *Phosphorus Sulfur Silicon Relat. Elem.* **183**, 1996–2005.

[bb3] Baur, W. H. (1974). *Acta Cryst.* B**30**, 1195–1215.

[bb4] Blessing, R. H. (1986). *Acta Cryst.* B**42**, 613–621.

[bb5] Brandenburg, K. & Putz, H. (2005). *DIAMOND* Crystal impact GbR, Bonn, Germany.

[bb6] Brown, I. D. (1976). *Acta Cryst.* A**32**, 24–31.

[bb7] Enraf–Nonius (1994). *CAD-4 EXPRESS* Enraf–Nonius, Delft, The Netherlands.

[bb8] Farrugia, L. J. (1997). *J. Appl. Cryst.* **30**, 565.

[bb9] Farrugia, L. J. (1999). *J. Appl. Cryst.* **32**, 837–838.

[bb10] Harms, K. & Wocadlo, S. (1996). *XCAD4* University of Marburg, Germany.

[bb11] Khemiri, H., Akriche, S. & Rzaigui, M. (2009). *Acta Cryst.* E**65**, o1152. 10.1107/S1600536809014536 PMC2977818 21583955

[bb12] Larafa, K., Mahjoub, A., Rzaigui, M. & Durif, A. (1997). *Eur. J. Solid State Inorg. Chem.* **34**, 481–494.

[bb13] Schülke, U. & Kayser, R. (1985). *Z. Anorg. Allg. Chem.* **531**, 167–175.

[bb14] Selmi, A., Akriche, S. & Rzaigui, M. (2009). *Acta Cryst.* E**65**, m1487. 10.1107/S1600536809044079 PMC2971134 21578209

[bb15] Sheldrick, G. M. (2008). *Acta Cryst.* A**64**, 112–122. 10.1107/S0108767307043930 18156677

